# La alfabetización en salud mental como pilar de la atención primaria

**DOI:** 10.1016/j.aprim.2026.103443

**Published:** 2026-02-03

**Authors:** Edwin Gustavo Estrada-Araoz

**Affiliations:** Universidad Nacional Amazónica de Madre de Dios, Puerto Maldonado, Perú

*Sr. Editor,*

Me dirijo a usted en relación con el editorial titulado «Atención primaria y salud mental: lecciones y desafíos en el mundo pospandemia», donde se reflexiona sobre la necesidad de integrar la salud mental en los servicios de primer nivel. Los autores destacan avances como la telemedicina, la capacitación profesional y la coordinación interinstitucional, todos esenciales para una atención integral^1^. No obstante, estimo que el trabajo podría enriquecerse al considerar un componente que reforzaría su propuesta: la alfabetización en salud mental (ASM).

La ASM se define como el conjunto de conocimientos y habilidades que permiten reconocer, comprender y gestionar los problemas psicológicos más comunes, así como buscar ayuda profesional de forma oportuna^2^. A pesar de su relevancia, este componente aún no se incorpora de manera sistemática en las políticas de salud pública ni en la práctica de la atención primaria. Se ha descrito que la ASM constituye un elemento esencial para promover la salud mental y reducir el estigma, al fortalecer la comprensión social y emocional, fomentar el apoyo entre pares y promover la búsqueda de ayuda oportuna tanto a nivel individual como comunitario^3^.

En muchos países latinoamericanos, los programas de salud mental continúan centrados en la atención clínica, con escasa inversión en educación psicosocial. La falta de información, los prejuicios culturales y la distancia geográfica dificultan que las personas reconozcan síntomas tempranos o busquen apoyo especializado. En este contexto, alfabetizar en salud mental significa mucho más que difundir conocimientos: implica construir espacios de diálogo, comprender las creencias locales sobre el sufrimiento psicológico y adaptar los mensajes educativos a los contextos lingüísticos y culturales.

La atención primaria constituye el escenario idóneo para liderar este cambio. Los profesionales de salud, por su cercanía con la comunidad, pueden promover estrategias de ASM en escuelas, asociaciones vecinales y centros laborales. Talleres sobre manejo del estrés, autocuidado o señales de alerta podrían contribuir a fortalecer la resiliencia individual y colectiva. Asimismo, incorporar la ASM en los programas de formación de médicos, enfermeros y agentes comunitarios fomentaría una atención más empática y centrada en la persona. De esta forma, los equipos de salud no solo atenderían el malestar psicológico, sino que también educarían para prevenirlo.

Por otro lado, la ASM puede aliviar la carga del sistema sanitario. Una ciudadanía informada tiende a consultar de forma más temprana, a seguir las indicaciones terapéuticas y a participar activamente en su propio proceso de recuperación. Esto reduce el estigma, mejora la adherencia y optimiza los recursos disponibles. Además, promueve la equidad, ya que brinda a los sectores más desfavorecidos la posibilidad de acceder al conocimiento y ejercer su derecho a la salud mental en condiciones más justas.

En conclusión, el artículo que motiva esta carta plantea con acierto la necesidad de fortalecer la atención primaria pospandemia. Sin embargo, la verdadera transformación requiere incorporar la ASM como un pilar estructural del sistema. Educar en salud mental no es una tarea complementaria, sino una estrategia de prevención, equidad y ciudadanía activa. Solo cuando las personas comprendan y valoren su bienestar emocional será posible consolidar sistemas sanitarios resilientes, inclusivos y sostenibles.

## Autoría

El manuscrito fue concebido, redactado y revisado en su totalidad por el autor, quien aprueba la versión final y asume plena responsabilidad por su contenido.

## Consentimiento informado

Dado que el contenido no incluye datos personales, historias clínicas ni testimonios de personas, no se requirió consentimiento informado.

## Financiación

La presente carta no ha recibido ningún tipo de financiación pública ni privada.

## Consideraciones éticas

Esta carta al editor no presenta resultados de investigación ni información proveniente de participantes humanos o animales. Por tal motivo, no fue necesaria la aprobación por parte de un comité de ética en investigación.

## Declaración sobre el uso de la IA generativa y de las tecnologías asistidas por la IA en el proceso de redacción

Durante la preparación de este manuscrito, el autor utilizó la herramienta de inteligencia artificial ChatGPT (OpenAI) exclusivamente como apoyo para la redacción y revisión del lenguaje. Posteriormente, el contenido fue revisado, editado y verificado de manera integral por el autor, quien asume la plena responsabilidad por la versión final del texto y su contenido científico.

## **Conflicto de intereses**

El autor declara que no existe ningún conflicto de intereses relacionado con el contenido de este manuscrito.

## References

[1] Pérez García MdP, Vázquez Canales LdM. (2025). Lo que la Organización Mundial de la Salud nos enseña sobre la salud mental en atención primaria. Aten Primaria..

[2] Jorm A.F. (2012). Mental health literacy: empowering the community to take action for better mental health. Am Psychol.

[3] Zeng S., Bailey R., Alkhamaiseh R.A., Peng S., Samsudin N. (2025). Mapping the big picture of mental health literacy in education: a bibliometric analysis. Humanit Soc Sci Commun..

